# Validity and reliability of single camera markerless motion capture systems with RGB-D sensors for measuring shoulder range-of-motion: a systematic review

**DOI:** 10.3389/fbioe.2025.1570637

**Published:** 2025-05-23

**Authors:** Unhyung Lee, Suji Lee, Sung-A Kim, Yohwan Kim, Seunghoon Lee

**Affiliations:** ^1^ Department of Acupuncture and Moxibustion Medicine, Kyung Hee University Medical Center, Seoul, Republic of Korea; ^2^ 20th Fighter Wing, Republic of Korea Air Force, Seosan, Republic of Korea; ^3^ Department of Acupuncture and Moxibustion, College of Korean Medicine, Kyung Hee University, Seoul, Republic of Korea; ^4^ Department of Clinical Korean Medicine, Graduate School, Kyung Hee University, Seoul, Republic of Korea

**Keywords:** shoulder motion, range of motion, RGB-D sensor, single camera markerless motion capture, systematic review

## Abstract

**Introduction:**

Assessing shoulder joint range-of-motion (ROM) is crucial for evaluating shoulder mobility but remains challenging due to its complexity. This review examined the potential of single-camera markerless motion capture systems with an RGB-depth (RGB-D) sensor for shoulder ROM measurements, focusing on their reliability and validity.

**Methods:**

We systematically searched nine databases through December 2022 for studies that evaluated the reliability and validity of single-camera markerless motion-capture systems in measuring simple (one-directional) and complex (multi-directional) shoulder movements. We extracted data on participant characteristics, device details, and measurement outcomes, and then assessed the methodological quality using the Consensus-Based Standards for the Selection of Health.

**Results:**

Of the 2,976 articles identified, 14 were included in this review. The findings indicate that intra-rater reliability findings across six studies were inconsistent, with simple movements like abduction and flexion demonstrating better reliability and less heterogeneity compared to complex movements. Validity assessments across 12 studies also showed inconsistency, with abduction and flexion measurements exhibiting higher validity than rotational movements. Studies focusing on simple movements reported good to excellent validity, particularly for abduction and flexion. Quality assessments using the COSMIN checklist revealed that the methodological quality varied across studies, ranging from inadequate to very good.

**Discussion:**

This systematic review suggests that RGB-D sensors show promise for measuring shoulder joint ROM, especially in simple movements like flexion and abduction. However, complex movements and inconsistencies limit their immediate clinical applicability, necessitating further high-quality research with advanced devices to ensure accurate and reliable assessments.

## 1 Introduction

The range of motion (ROM) is essential for assessing joint mobility as it indicates the current pathological or physiological state of a joint. Assessing shoulder motion can help differentiate between various shoulder disorders, including rotator cuff tears, adhesive capsulitis, and impingement syndrome (Lee et al., 2021). Additionally, the effect of a treatment can be ascertained by comparing the ROM before and after. Therefore, measuring shoulder ROM measurement is important in clinical practice.

The shoulder joint complex comprises the acromioclavicular, glenohumeral, scapulothoracic, and sternoclavicular joints; shoulder motion involves the coordination of all these joints. Therefore, performing consistent shoulder motion measurements is complex and challenging, necessitating an accurate and consistent measurement method (Tondu, 2007; Muir et al., 2010).

Goniometry is the most commonly used method for measuring joint ROM. However, its low inter-rater reliability and measurement variability make its application in clinical settings challenging (Beshara et al., 2021). Although an alternate 3D-marker-based motion tracking system are available, they are expensive and requires a large space, considerable time, and experienced clinicians. These limitations render traditional motion-capture systems unsuitable for routine clinical use (Reither et al., 2018).

In comparison, markerless motion-capture systems, which are low-cost and comprise RGB and depth cameras, offer a good alternative for upper-limb assessment. These systems emit light from a source, measure the backscatter using a depth camera, and translate the delay into a distance value (Tölgyessy et al., 2021). Initially commercialized as an add-on (Kinect V1) for the Xbox 360 console (Microsoft Corp., Redmond, WA) in 2009, this technology has been adapted for various applications, including kinematic motion analysis (Han et al., 2013). Since then, several manufacturers have developed RGB-depth (RGB-D) sensors. Microsoft released Kinect V2 in 2013; Orbbec released Astra Pro (Orbbec, Troy, MI) in 2016; Intel released RealSense (Intel Corp., Santa Clara, CA) in 2017; and Microsoft subsequently launched Azure Kinect (Kinect V4) in 2019. These systems can detect joint orientation and track joint and skeletal positions, allowing joint motion and kinematic analyses without significant space or financial constraints (Beshara et al., 2021). Hence, they can be considered potential alternatives to goniometry and traditional motion-capture systems for upper-limb assessment.

The reliability and validity of measurements obtained through ROM assessment devices are critical for their clinical use (Streiner et al., 2015). Although several studies (Muaremi et al., 2019; Kuster et al., 2016; Cai et al., 2019) have reported the validity and reliability of markerless motion-capture systems for measuring ROM, their measurement characteristics vary, and results are inconsistent. Additionally, only one systematic review on shoulder ROM using a markerless motion-capture system exists, which evaluate using the intraclass correlation coefficient (ICC) but did not assess validity (Beshara et al., 2021). Therefore, this study aimed to systematically review the reliability and validity of single-camera markerless motion-capture system using an RGB-D sensor to measure shoulder ROM.

## 2 Material and methods

This systematic review was designed based on the Preferred Reporting Items for Systematic Reviews and Meta-Analysis Protocols (PRISMA-P) 2020 statement (Page et al., 2021). The review protocol was registered online at PROSPERO (CRD42023395441) and has been published previously (Lee et al., 2023).

### 2.1 Study types

We included studies that measured shoulder ROM using a single-camera markerless motion-capture system with an RGB-D sensor and assessed the intra-rater reliability, inter-rater reliability, and validity of the device. Studies employing Microsoft Kinect V1 were excluded because Kinect V1 measures depth using the pattern projection principle, whereas Kinect V2 and Azure Kinect use the continuous wave intensity modulation approach, which is primarily used in time-of-flight cameras (Tölgyessy et al., 2021). Additionally, several studies have found that the Kinect V2 offer more reliable ROM measurements (Reither et al., 2018; Foreman and Engsberg, 2020). Case studies, review articles, studies without full text, and studies that did not measure shoulder joints by angle were excluded.

### 2.2 Participants

Studies on healthy participants of all ages, as well as those shoulder or upper-limb motor disorders were included.

### 2.3 Outcome measurements

This study primarily assessed the intra- and inter-rater reliabilities and validity of markerless motion-capture systems used for measuring shoulder movements. One-directional movements (e.g., flexion, extension, abduction, adduction, external rotation, or internal rotation) are defined as simple movements, whereas multi-directional movements (e.g., wheelchair transfer or hair combing) are defined as complex movements. The results for these movements are included in the main outcomes. Both active and passive ROM values were considered as the main outcomes. For validity evaluation, studies were required to compare measurements from markerless motion-capture systems against a reference standard. Acceptable reference standards included marker-based motion capture systems, goniometers, or other validated motion measurement devices. No restrictions were placed on the type of reference standard, but the comparison method had to be clearly described.

### 2.4 Data sources and search methods

Two independent reviewers searched the MEDLINE, EMBASE, Cochrane Library, Cumulative Index to Nursing and Allied Health Literature (CINAHL) using EBSCO, IEEE Xplore, China National Knowledge Infrastructure (CNKI), KoreaMed, Korean Studies Information Service System (KISS), and Research Information Sharing Services (RISS) databases. The search string comprised three terms: RGB-D sensor (e.g., Kinect, RGB-D camera, or infrared), shoulder (e.g., shoulder, upper limb, or upper extremity), and ROM (e.g., ROM or kinematics). Details of the search string are presented in the Supplementary Material. The search included all studies published until December 2022. If a study meeting the inclusion criteria was found outside the search area, it was included with the consent of the two reviewers.

### 2.5 Data extraction and quality assessment

Two independent reviewers extracted data, including basic information (author names and year of publication), population characteristics (mean age, sex, height, mass, and sample size), device details (type and description of the camera and software), and study design (measurement methods, movements performed, participant position, results of intra- and inter-rater reliability, validity, statistical methods, number of raters, number of sessions, and session interval). To assess the reliability and validity of the studies, two reviewers independently evaluated them using three metrics from the Consensus-based Standards for the Selection of Health Measurement Instruments (COSMIN) checklist: reliability, measurement errors compared to other outcome measurement instruments, and criterion validity (Mokkink et al., 2021). COSMIN is generally used for patient-reported outcome measures; therefore, an extended version is recommended for assessing reliability and measurement errors. In this study, nine standards for reliability, eight standards for measurement errors, and three standards for validity were assessed on a four-point scale (very good, adequate, doubtful, and inadequate), and the method with the worst score was used for grading. Disagreements regarding eligibility were resolved through discussions with a third reviewer.

### 2.6 Data synthesis

The principal analysis focused on intra- and inter-rater reliability and validity of single-camera markerless motion-capture systems using RGB-D sensors to measure shoulder joint angles. Subgroup analyses examined study design factors that might influence reliability and validity scores, including system type (e.g., Kinect V2, Azure Kinect, or RealSense), movement direction (flexion, extension, abduction, adduction, external rotation, or internal rotation), and movement complexity (simple or complex movements). Correlation values were interpreted based on the following criteria: less than 0.5 indicated poor, 0.5–0.75 indicated moderate, 0.75–0.9 indicated good, and greater than 0.90 indicated excellent reliability (Puh et al., 2019). Various correlation coefficients were extracted and reported in the following order: ICC, concordance correlation coefficient (CCC), and correlation coefficient (CC). ICC, CCC, and CC reflect the strength of association between repeated measurements, whereas the limits of agreement (LOA), typically obtained through Bland-Altman analysis, assess the absolute agreement between measurements. A LOA within ±10° was considered clinically acceptable based on previous studies (Rigoni et al., 2019).

## 3 Results

### 3.1 Study selection

**FIGURE 1:**
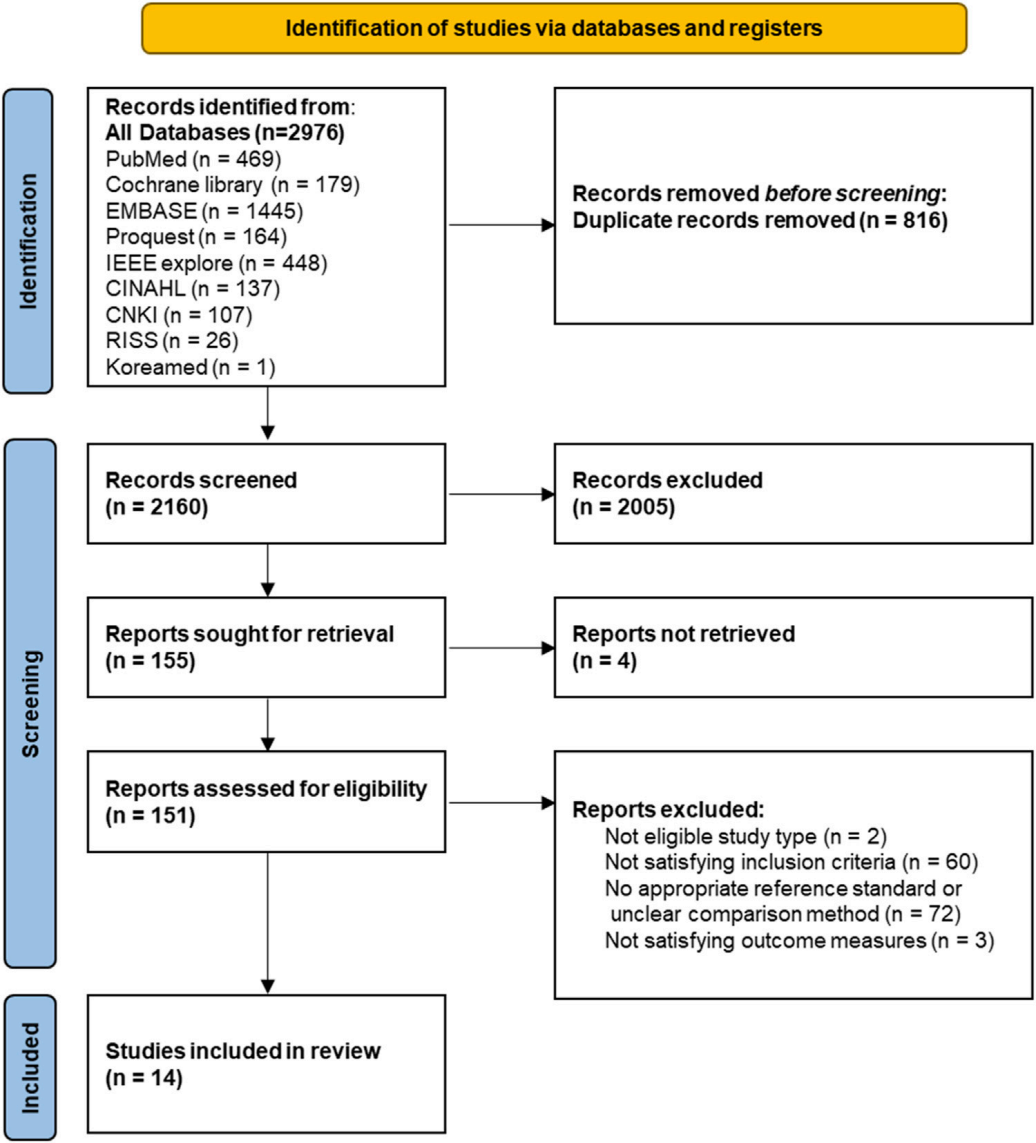
Flowchart of study selection according to the PRISMA diagram.

We identified 2,976 potential studies from nine databases. After removing 816 duplicate records 2,160 studies remained for screening. Two reviewers independently screened the titles and abstracts of these studies. We excluded 2,005 studies based on unsuitable titles and abstracts, and four studies were unobtainable. This left 151 studies for full-text assessment. Of these, 137 were excluded for not involving an eligible study type, not meeting the inclusion criteria, lacking an appropriate reference standard or a clear comparison method, or not reporting relevant outcome metrics such as reliability or validity. Finally, 14 studies were included in this systematic review (Figure 1).

### 3.2 Characteristics of included studies

The included studies are summarized in Tables 1 and 2. Of the 14 studies, 12 used Kinect V2 (Microsoft Corp, Redmond, WA), three used Azure Kinect (Microsoft Corp, Redmond, WA), and two used RealSense (Intel Corp, Santa Clara, CA) as the single-camera markerless motion-capture system. Studies using Kinect V2 have been conducted since 2016, while those employing Azure Kinect and RealSense have been conducted since 2022. Five studies (De Paolis and De Luca, 2020; Çubukçu et al., 2020; Koontz et al., 2022; Foreman and Engsberg, 2020; Cai et al., 2019) assessed only intra-rater reliability, while one study (Özsoy et al., 2022) assessed both intra- and inter-rater reliabilities. Twelve studies assessed the validity; of these, 10 used marker-based motion-capture systems as the reference standard. The Vicon motion analysis system (Oxford Metrics, Oxford, United Kingdom) was the most used (Oxford Metrics, Oxford, United Kingdom) was the most used (Faity et al., 2022; Cai et al., 2019; Zulkarnain et al., 2017; Kuster et al., 2016), while other studies employed systems such as BTS smart Dx-100 (BTS Bioengineering, Milan, Italy) (Özsoy et al., 2022), Qualisys Track manager (Qualisys Ltd., Sweden) (Jo et al., 2022), MAC Eagle Digital Cameras (Motion Analysis Corp., Santa Rosa, CA, United States) (Foreman and Engsberg, 2020), and Optotrack Certus System (Northern Digital, Canada) (Xu et al., 2017). Three studies (Gauci et al., 2023; Mangal and Tiwari, 2020; Çubukçu et al., 2020) used a goniometer as the reference standard. Among them, one study (Gauci et al., 2023) used visual estimation by experts, while another (Çubukçu et al., 2020) used both digital and manual goniometers. One study (Muaremi et al., 2019) used angle assessments from images through four-color cameras as the reference standard.

**TABLE 1:** Summary of included studies.

Author	Movement requested	Position (patient/device)	Session	Assessor	Time interval
Simple movement					
Kuster et al. (2016)	F/Abd/ER-EF	Standing, Seated/Frontal	5 exercises × 5 rep × 2 (standing, seated)	Not reported	Not reported
Zulkarnain et al. (2017)	F/Abd/ER-EF/IR-EF	Seated/Frontal	4 pose × 10 rep	Not reported	Not reported
Muaremi et al. (2019)	F/E/Abd/ER-EF/IR-EF (4 different angles sequentially larger)	Standing/Frontal	Not reported	Not reported ground truth – 2 surgeons	3 of 5 separate testing session time in 3 days
Çubukçu et al. (2020)	F/E/Abd/ER-EF/IR-EF	Standing/Frontal	3 (3 rep)	Not reported	Not reported
De Paolis and De Luca (2020)	Abd (2 height)	Standing/Frontal	2 height × 30 rep	Not reported	Not reported
Mangal and Tiwari (2020)	F/E/Abd/ER-EF	Not reported	1	Not reported	Not reported
Jo te al., 2022	F/Abd	Standing/Frontal	5 rep	Not reported	Not reported
Özsoy et al. (2022)	F/Abd/ER-EF/IR-EF/ER-EE/IR-EE	Standing/Frontal	5 rep	2	Not reported
Gauci et al. 2023	F/E/Abd/Add/ER-EF/ER-Abd/IR-Abd	Standing/Frontal (Abd/Add/ER/IR), lateral (F/E)	1 (depth 1 session visual, goniometric 1 session simultaneously)	2 shoulder expert surgeons	2 shoulder expert surgeons
Xu et al. (2017)	10 min computer task	Seated/Frontal	10 min task × 3	Not reported	Not reported
Complex movement					
Cai et al. (2019)	1) Hand to contralateral shoulder 2) Hand to mouth or drinking 3) Combing hair 4) Hand to back pocket	Standing/Frontal	2 × 4 task × at least 5 rep	Not reported	Not reported
Foreman and Engsberg (2020)	Reaching toward a target (sagittal, scaption, frontal plane/non-extended, extended (20 cm beyond arm length)	Seated/Frontal	2 days × 5 rep × 4 set × 3 direction × 2 condition	Not reported	3-5 minutes between set different day
Faity et al. (2022)	Reaching a table tennis ball	Seated/Frontal	5 rep x 2 (trunk use, restrained trunk)	Not reported	Not reported
Koontz et al. (2022)	Transfer from wheelchair to the transfer surface using their habitual technique	Seated/Frontal	Phase 1 (disabled): 5 rep Phase 2 (healthy volunteers): 10 rep	Not reported	3-5 minutes (more time if needed)

Abbreviations: F: flexion; E, extension; Abd, abduction; Add, adduction; ER-EF, external rotation-elbow flexed; IR-EF, internal rotation-extended flexed; ER-EE, external rotation-elbow extended; IR-EE, internal rotation-elbow extended; ER-Abd, external rotation-shoulder joint abduction at 90°; IR-Abd, internal rotation-shoulder joint abduction at 90°; rep, repetition.

**TABLE 2:** Summary of characteristics of included studies.

Author	Sample size	Age		Height/weight (cm/kg)	Sex (male/female)	Inclusion	Device	Comparison (as gold standard)	Device height/Distance (meter)	Software (SDK/filter)
Simple movement										
Kuster et al. (2016)	20	33 ± 9		173 ± 8.4/65.9 ± 10.6	10/10	Healthy volunteers	Kinect V2	Marker-based motion tracking system (Vicon system)	1.2/2.5	SDK 2.0/Butterworth 2nd-order LPF (cut-off 2 Hz)
Zulkarnain et al. (2017)	10	25.8 ± 4.6		Not reported	10/0	Healthy volunteers	Kinect V2	Marker-based motion tracking system (Vicon system)	Not reported/2	SDK 2.0/Not reported
Muaremi et al. (2019)	20	Not reported		Not reported	Not reported	Healthy volunteers	Kinect V2	4 color cameras	Not reported	Not reported/Not reported
Çubukçu et al. (2020)	40	22.1 ± 3.1		170.9 ± 8.8/Not reported	22/18	Healthy volunteers	Kinect V2	Digital goniometer Goniometer	Not reported/2	SDK 2.0/Not reported
De Paolis and De Luca (2020)	2	Not reported		185, 156/Not reported	1/1	Not reported	Kinect V2	Not reported	0.8/2.1	SDK 2.0/Not reported
Mangal and Tiwari (2020)	Not reported	Not reported		Not reported	Not reported	Not reported	Kinect V2	Goniometer	Not reported	SDK 2.0/Custom kinematics-based filter
Jo et al. 2022	12 (24 shoulder)	23.2 ± 2.0		171.9 ± 8.5/61.2 ± 12.8	5/5	Healthy volunteers	Azure Kinect	Marker-based motion tracking system (Qualisys Track Manager)	Not reported	Azure Kinect SDK 1.3.0/Butterworth 4th-order LPF (cut-off 6 Hz)
Özsoy et al. (2022)	10 (reliability)	27.9 ± 4.8		Body mass index 24 ± 4	10/10	Healthy volunteers	Kinect V2 Azure Kinect	Marker-based motion tracking system (BTS smart DX-100)	0.8/1.5	iPiSoft suite (SDK-equivalent platform)/Not reported
	20 (validity)									
Gauci et al. (2023)	30	33 ± 7		Not reported	20/10	Healthy volunteers	Reasense D435	Goniometer, visual estimation	1.5/3	Not reported/Not reported
Complex movement										
Xu et al. (2017)	11	26.5 ± 9.2		171 ± 0.1/70.3 ± 10.9	6/5	Healthy volunteers	Kinect V2	Marker-based motion tracking system (Optotrack Certus system)	Adjusted to upper body/0.75	SDK 2.0/Not reported
Cai et al. (2019)	10	24.6 ± 2.8		174.1 ± 4.4/67.2 ± 4.2	10/0	Healthy volunteers	Kinect V2	Marker-based motion tracking system (Vicon system)	0.8/2	Not reported/Butterworth LPF (cut-off 6 Hz)
Foreman and Engsberg (2020)	5	24.8		Not reported	2/3	Healthy volunteers	Kinect V2	Marker-based motion tracking system (MAC eagle digital cameras)	1.2/2	SDK 2.0/Butterworth 6th-order LPF (cut-off 6 Hz)
Faity et al. (2022)	26	21 ± 3		173 ± 9/66.9 ± 9.3	12/14	Healthy volunteers	Kinect V2	Marker-based motion tracking system (Vicon system)	1.4/1.5	SDK 2.0/Butterworth 2nd-order LPF (dual-pass)
Koontz et al. (2022)	Phase 1	30	56.6	Not reported	26/4	Wheel chair user	Kinect V2 Azure Kinect	Not reported	Not reported/2 (Kinect V2) 1 (Realsense)	Windows Kinect SDK pyKinectAzure Nuitrack SDK/Not reported
	Phase 2	7	29.4		3/4	Healthy volunteers	Kinect V2 RealsenseD435	Not reported	Not reported/2.13	

Abbreviations: SDK: software development kit; LPF: low-pass filter.

All 14 studies included healthy volunteers as participants, except one study (Koontz et al., 2022), which assessed validity and reliability for individuals with disabilities involving low extremity impairments who used wheelchairs for mobility tasks. Three studies (Özsoy et al., 2022; De Paolis and De Luca, 2020; Çubukçu et al., 2020) assessed simple movements such as flexion, extension, abduction, adduction, external rotation, and internal rotation, while four studies (Koontz et al., 2022; Faity et al., 2022; Foreman and Engsberg, 2020; Cai et al., 2019) assessed complex movements, including wheelchair transfers (Koontz et al., 2022), hand-to-mouth tasks (Cai et al., 2019), reaching a table tennis ball (Faity et al., 2022), and reaching a target (Foreman and Engsberg, 2020 The cameras were positioned at a height of 0.8–1.5 m and a distance of 1.five to three m from the participant. In most studies, the camera was placed in front of the participant. However, one study (Gauci et al., 2023) positioned it laterally to assess flexion and extension movements, and another (Mangal and Tiwari, 2020) did not report the camera placements.

Measurements were taken in two postures: sitting and standing. Seven studies (Muaremi et al., 2019; Özsoy et al., 2022; De Paolis and De Luca, 2020; Çubukçu et al., 2020; Jo et al., 2022; Gauci et al., 2023; Cai et al., 2019) used standing postures, five studies (Zulkarnain et al., 2017; Foreman and Engsberg, 2020; Koontz et al., 2022; Faity et al., 2022; Xu et al., 2017) used sitting posture, and one study (Kuster et al., 2016) included both.

The criteria for assessing study quality based on sample size were established based on a previous review (Beshara et al., 2021). Studies with sample sizes of one were deemed inappropriate, <10 were considered doubtful, 10–30 were considered adequate, and ≥30 were considered very good. Three studies (Gauci et al., 2023; Koontz et al., 2022; Çubukçu et al., 2020) had sample sizes of ≥30, four studies (Koontz et al., 2022; De Paolis and De Luca, 2020; Foreman and Engsberg, 2020; Mangal and Tiwari, 2020) included 2–10 participants, one study (Mangal and Tiwari, 2020) did not report the sample size, and the remaining studies had sample sizes of 10–30.

In addition to hardware specifications, the software environments, including the software development kits (SDKs) and filtering methods, were also summarized in each study. Most studies that used Kinect V2 employed Kinect for Windows SDK 2.0. Filtering methods were not always specified, but when reported, a Butterworth low-pass filter was commonly used.

### 3.3 Reliability

**FIGURE 2:**
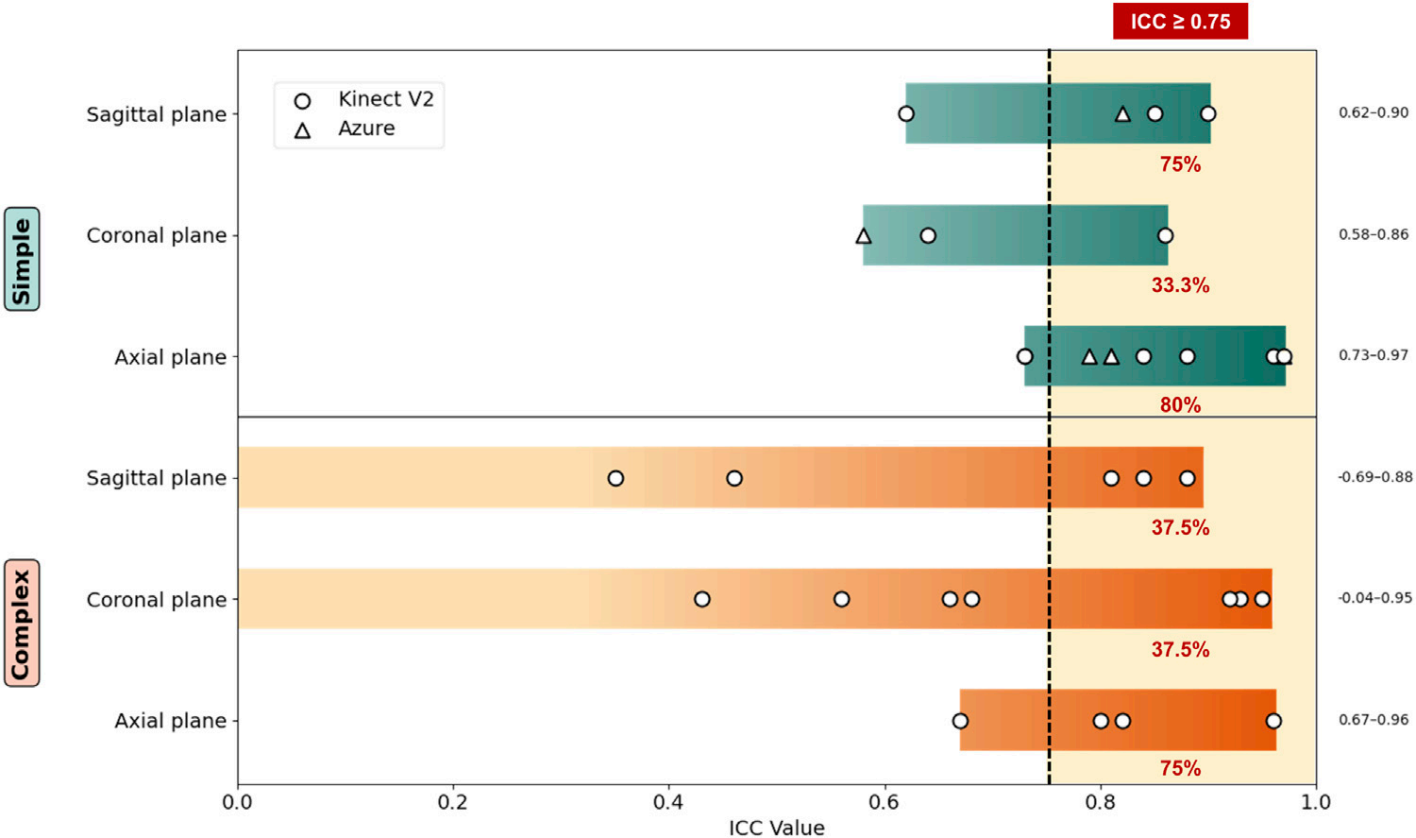
Reliability of single-camera markerless motion capture systems using an RGB-D sensor to measure shoulder ROM. ICC: intraclass correlation. Movement directions are grouped by anatomical planes: sagittal plane (flexion/extension), coronal plane (abduction/adduction), and axial plane (internal/external rotation). The percentage next to each movement group indicates the proportion of measurements with an ICC ≥0.75. A vertical dashed line denotes the ICC ≥0.75 threshold for good-to-excellent reliability.

The intra- and inter-rater reliabilities of the reviewed studies are presented in Table 3. Considerable variability was observed among the six studies assessing intra-rater reliability. One study (Foreman and Engsberg, 2020) used a Bland–Altman plot to report reliability, finding that the LOA for all movements exceeded 10°. Another study (De Paolis and De Luca, 2020) reported reliability using the coefficient of variation, whereas five reported reliability using correlation. Two studies (Özsoy et al., 2022; Çubukçu et al., 2020) evaluating simple movements reported moderate-to-excellent reliability, whereas three studies (Koontz et al., 2022; Foreman and Engsberg, 2020; Cai et al., 2019) assessing complex movements demonstrated heterogeneous results. One study (Koontz et al., 2022) that analyzed wheelchair transfers reported relatively low reliability (poor-to-moderate) for people with disabilities compared with healthy individuals. Another study (Cai et al., 2019) observed poor-to-moderate reliability for several complex movements, particularly for the “combing hair” task, which negatively affected the overall reliability. Conversely, one study (Foreman and Engsberg, 2020), found that abduction demonstrated relatively good reliability, while flexion and scaption movements showed poor reliability. Overall, the reliability for simple movements was superior to that for complex movements (Figure 2). Among simple movements (Özsoy et al., 2022; Çubukçu et al., 2020), the highest reliability was obtained for internal rotation movements. Although there were slight differences for other movements, flexion showed good overall reliability and abduction showed moderate-to-good reliability.

**TABLE 3:** Reliability scores of single camera markerless motion capture systems using RGB-D sensors for shoulder ROM measurements.

Author	Measure type	Device	F	E	Abd	Add	ER	IR
Simple movement								
Çubukçu et al. (2020)	Intra-rater ICC	Kinect V2	0.85	0.62	0.86	—	(EF) 0.84	(EF) 0.97
De Paolis and De Luca (2020)	Intra-rater CV	Kinect V2	—	—	(Height 1.48 m) 0.021∼0.029 (Height 1.73 m) 0.017∼0.018	—	—	—
Özsoy et al. (2022)	Intra-rater ICC	Kinect V2	0.90	—	0.64	—	(EF) 0.73 (EE) 0.85	(EF) 0.96 (EE) 0.88
		Azure	0.82	—	0.58	—	(EF)0.81 (EE) 0.94	(EF) 0.97 (EE) 0.79
	Inter-rater ICC	Kinect V2	0.84	—	0.78	—	(EF) 0.56 (EE) 0.47	(EF) 0.65 (EE) 0.52
		Azure	0.64	—	0.93	—	(EF) 0.79 (EE) 0.82	(EF) 0.89 (EE) 0.70
Complex movement								
Cai et al. (2019)	Intra-rater ICC	Kinect V2	Hand to the contralateral shoulder					
			(F-E) 0.88		(Abd-Add) 0.68		(ER-IR) 0.96	
			Hand to mouth or drinking					
			(F-E) 0.81		(Abd-Add) 0.95		(ER-IR) 0.80	
			Combing hair					
			(F-E) 0.35		(Abd-Add) 0.43		(ER-IR) 0.67	
			Hand to back pocket					
			(F-E) 0.84		(Abd-Add) 0.92		(ER-IR) 0.82	
Foreman and Engsberg (2020)	Intra-rater ICC	Kinect V2	(FR) 0.46 (Extended FR) −0.23 (SR) −0.22 (Extended SR) −0.69	—	(LR) 0.93 (Extended LR) 0.66 (SR) 0.56 (Extended SR) −0.04	—	—	—
	Intra-rater LOA	Kinect V2	(FR) −16.8∼20.8 (Extended FR) −30.9∼45.1 (SR) −26.5∼20.5 (Extended SR) −18.8∼21.1	-	(LR) −10.4∼17.7 (Extended LR) −11.1∼26.2 (SR) −32.3∼22.4 (Extended SR) −20.4∼20.1	—	—	—
Koontz et al. (2022)	Intra-rater ICC	Kinect V2	Transfer from wheelchair to the transfer surface Average joint angle between trunk and the upper arm		(Disabled) 0.60, (Healthy volunteers) 0.89			
		RealSesnse			(Disabled) 0.38			
		Azure			(Healthy volunteers) 0.92			

Abbreviations: F, flexion; E, extension; Abd, abduction; Add, adduction; ER, external rotation; IR, internal rotation; ICC, intraclass correlation; EF, elbow flexed; EE, elbow extended; LOA, limits of agreement; CV, coefficient of variation; FR, forward reach; SR, scaption reach (45° angle); LR, lateral reach; “—” indicates results not reported.

Regarding device-specific differences, the only study using Azure Kinect (Özsoy et al., 2022) reported no significant difference in reliability between Azure Kinect and Kinect V2; however, Azure Kinect showed superior performance in rotation measurements.

Only one study (Özsoy et al., 2022) assessed inter-rater reliability, reporting moderate-to-good reliability for Kinect V2 and moderate-to-excellent reliability for Azure Kinect. Notably, Azure Kinect performed better in external rotation measurements.

### 3.4 Validity

The validity scores are summarized in Table 4, showing a wide range of results across studies. Two studies (Çubukçu et al., 2020; Zulkarnain et al., 2017) that employed Bland–Altman plot reported clinically acceptable discrepancies, the remaining five studies (Gauci et al., 2023; Jo et al., 2022; Özsoy et al., 2022; Faity et al., 2022; Kuster et al., 2016; Foreman and Engsberg, 2020) reported discrepancies exceeding 10°.

**TABLE 4:** Validity scored of single camera markerless motion capture systems using RGB-D sensor to measure shoulder ROM.

Author	Measure type	Device	Comparator	F	E	Abd	Add	ER	IR
Simple movement									
Kuster et al. (2016)	CC	Kinect V2	MTS	(Seated) 0.93 (Stand) 0.97	—	(Seted) 0.99 (Stand) 0.99	—	(Seated) 0.96 (Stand) 0.98	—
	LOA			(Seated) −22.0∼36.4 (Stand) 2.9∼20.7	—	(Seated) −12.9∼7.1 (Stand) −10.7∼11.4	—	(Seated) −29.2∼30.4 (Stand) −11.2∼17.6	—
Zulkarnain et al. (2017)	CC	Kinect V2	MTS	0.97	—	0.99	—	(EF) 0.94	(EF) 0.97
	LOA			−8.4∼7.3	—	−7.1∼7.8	—	(EF) −8.0∼7.4	(EF) −8.1∼3.6
Muaremi et al. (2019)	CC	Kinect V2	Camera	0.99	0.97	0.98	—	(IR) 0.99	
Çubukçu et al. (2020)	LOA	Kinect V2	Digital goniometer	−9.03∼5.68	−0.28∼0.33	−2.63∼4.83	—	(EF) −6.11∼5.36	(EF) −18.3∼7.6
			Goniometer	−9.88∼4.23	−1.69∼1.49	−4.86∼5.51	—	(EF) −7.5∼6.55	(EF) −21.42∼8.07
Mangal and Tiwari (2020)	ICC	Kinect V2	Goniometer	0.96	0.95	0.98	—	(EF) 0.98	—
Özsoy et al. (2022)	ICC	MTS	Kinect V2	0.86	—	0.78	—	(EF) 0.60 (EE) 0.38	(EF) 0.74 (EE) 0.49
			Azure	0.82	—	0.79	—	(EF) 0.66 (EE) 0.67	(EF) 0.70 (EE) 0.75
	LOA	MTS	Kinect V2	−17.2∼11.9	—	−36.9∼11.8	—	(EF) −29.9∼17.9 (EE) −16.2∼24.1	(EF) −15.3∼27.3 (EE) −1.7∼36.5
			Azure	−20.2∼12.3	—	−31.9∼12.7	—	(EF) −31.1∼16.4 (EE) −12.2∼19.4	(EF) −19.1∼28.2 (EE) 3.4∼32.1
Jo et al. (2022)	ICC	Azure	MTS	Right 0.68 Left 0.74	—	Right 0.92 Left 0.91	—	—	—
	LOA			Right −48.8∼38.3 Left −44.3∼32.4	—	Right −9.9∼27.5 Left −15.8∼29.2	—	—	—
Gauci et al. (2023)	CCC	Realsense	Goniometer	0.97	0.90	0.98	0.91	(EF) 0.82 (Abd) 0.94	(Abd) 0.87
			Visual estimation	0.95	0.85	0.97	0.86	(EF) 0.82 (Abd) 0.91	(Abd) 0.91
	LOA	Realsense	Goniometer	−17.6∼15.3	−12.2∼7.08	−14.6∼13.9	−11.4∼9.13	(EF) −17.9∼32.9 (Abd) −9.4∼20.4	(Abd) −14.1∼24.2
			Visual estimation	−22.7∼12.5	−10.6∼13.9	−18.8∼10.9	−14.6∼−11.0	(EF) −13.3–10.9 (Abd) −12.1∼12.3	(Abd) −6.4∼10.7
Complex movement									
Xu et al. (2017)	CCC	Kinect V2	MTS	Computer task					
				(F-E) R 0.40, L 0.40		(Abd-Add) R 0.28, L 0.17		(ER-IR) R 0.32, L 0.06	
	CC			(F-E) R 0.68, L 0.75		(Abd-Add) R 0.84, L 0.55		(ER-IR) R 0.57, L 0.16	
Cai et al. (2019)	CC	Kinect V2	MTS	Hand to contralateral shoulder					
				(F-E) 0.98		(Abd-Add) 0.99		(ER-IR) 0.74	
				Hand to mouth or drinking					
				(F-E) 0.91		(Abd-Add) 0.95		(ER-IR) 0.33	
				Combing hair					
				(F-E) 0.20		(Abd-Add) 0.65		(ER-IR) 0.77	
				Hand to back pocket					
				(F-E) 0.91		(Abd-Add) 0.96		(ER-IR) −0.10	
Foreman and Engsberg (2020)	CC	Kinect V2	MTS	(FR) 0.24 (Extended FR) 0.36 (SR) 0.80 (Extended SR) 0.66	—	(LR) 0.96 (Extended LR) 0.89 (SR) 0.97 (Extended SR) 0.91	—	—	—
	LOA			(FR) −31.2∼−2.5 (Extended FR) −37.5∼−5.1 (SR) −29.2∼−3.3 (Extended SR) −37.4∼−16.0	—	(LR) −16.3∼−4.9 (Extended LR) −24.2∼−7.1 (SR) −26.4∼−11.7 (Extended SR) −33.9∼−22.1	—	—	—
Faity et al. (2022)	ICC	Kinect V2	MTS	Seated hand-reaching tasks while holding a dumbbell					
				0.50	—	0.29	—	—	—
	LOA			−15.68∼28.90	—	−19.75∼25.08	—	—	—

Abbreviations: F, flexion; E, extension; Abd, abduction; Add, adduction; ER, external rotation; IR, internal rotation; CCC, concordance correlation coefficient; ICC, intraclass correlation; CC, correlation coefficient; LOA, limits of agreement; MTS, Marker-based motion tracking system; EF, elbow flexed; EE, elbow extended; FR, forward reach; SR, scaption reach (45° angle); LR, lateral reach; R, right; L, left; “—” indicates results not reported.

**FIGURE 3:**
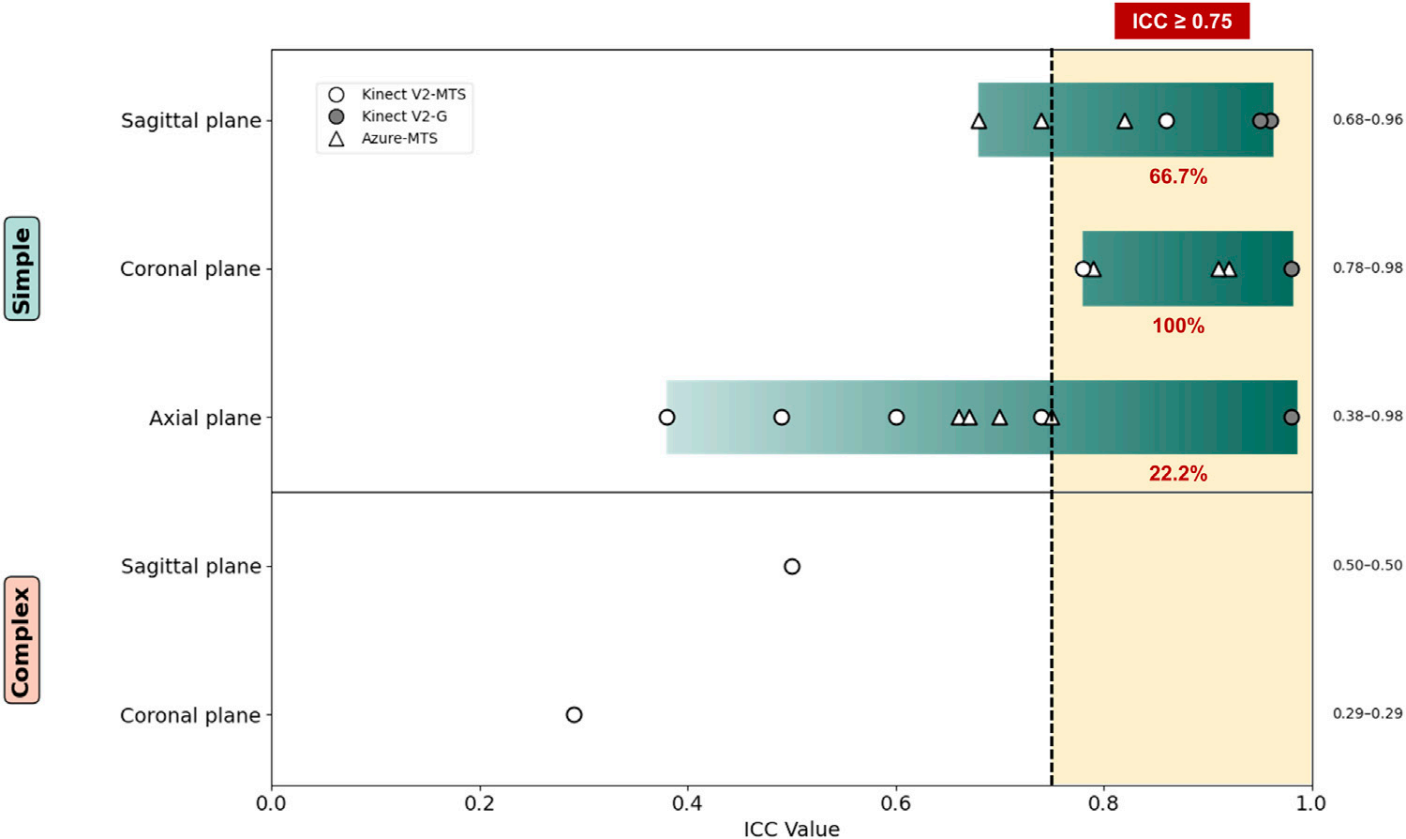
Validity of single-camera markerless motion capture systems using an RGB-D sensor to measure shoulder ROM ICC: intraclass correlation. Movement directions are grouped by anatomical planes: sagittal plane (flexion/extension), coronal plane (abduction/adduction), and axial plane (internal/external rotation). The percentage next to each movement group indicates the proportion of measurements with an ICC ≥0.75. A vertical dashed line denotes the ICC ≥0.75 threshold for good-to-excellent validity.

Most other studies assessed validity through correlation, as illustrated in Figure 3. The overall validity scores across the studies were heterogeneous. Five studies (Kuster et al., 2016; Zulkarnain et al., 2017; Mangal and Tiwari, 2020; Muaremi et al., 2019; Gauci et al., 2023) reported good-to-excellent, one (Jo et al., 2022) reported moderate-to-excellent, one (Faity et al., 2022) reported poor-to-moderate, and one (Xu et al., 2017) reported poor levels of validity. Additionally, other studies reported varying levels, ranging from poor to good (Özsoy et al., 2022) and poor to excellent (Foreman and Engsberg, 2020; Cai et al., 2019), depending on the specific movement assessed.

In general, studies focusing on simple movements demonstrated better validity scores than those assessing complex movements. Four studies (Xu et al., 2017; Cai et al., 2019; Foreman and Engsberg, 2020; Faity et al., 2022) that analyzed complex movements reported significant variability in results depending on the movement type, leading to poor overall validity scores. Conversely, studies examining simple movements exhibited less heterogeneity, with only one study (Özsoy et al., 2022) reporting poor validity. This result was specific to measurements of external rotation with the elbow extended; however, except abduction, measurements of other movements resulted in above-moderate levels of validity.

Among the various movements, abduction and flexion consistently yielded higher validity scores. All studies (Gauci et al., 2023; Özsoy et al., 2022; Mangal and Tiwari, 2020; Çubukçu et al., 2020; Muaremi et al., 2019; Zulkarnain et al., 2017; Kuster et al., 2016; Cai et al., 2019; Xu et al., 2017) that measured flexion, abduction, and rotation reported higher or similar validities for flexion and abduction compared to rotation. Studies employing Bland–Altman plots showed that approximately half (Gauci et al., 2023; Çubukçu et al., 2020; Zulkarnain et al., 2017) had around 10° LOA for abduction and flexion, indicating their clinical usability. Furthermore, of the studies presenting results as correlations, 72.7% (8/11) reported good or higher validity scores for abduction, 54.5% (6/11) for flexion, and 57.1% (4/7) for external and internal rotations.

Regarding the device used, most studies used Kinect V2, two studies (Jo et al., 2022; Özsoy et al., 2022) used Azure Kinect, and only one study (Gauci et al., 2023) used RealSense. One study (Özsoy et al., 2022) compared Azure Kinect and Kinect V2, reporting similar validity scores for overall movement measurements but superior validity for rotation measurements with Azure Kinect. Another study using Azure Kinect (Jo et al., 2022) obtained moderate validity for flexion and excellent validity for abduction, comparable to Kinect V2. Additionally, the study using RealSense (Gauci et al., 2023) exhibited good validity scores for rotation measurements and excellent validity for flexion and abduction.

### 3.5 Methodological evaluation of the measurement properties

A methodological evaluation was conducted using the COSMIN checklist. Of the nine studies assessed using this checklist (Table 5), four (Koontz et al., 2022; Foreman and Engsberg, 2020; Çubukçu et al., 2020; Cai et al., 2019) were rated as adequate, two (Özsoy et al., 2022; Mangal and Tiwari, 2020) as doubtful, and three (De Paolis and De Luca, 2020; Zulkarnain et al., 2017; Kuster et al., 2016) as inadequate. Additionally, three studies did not express the results using ICC and five studies (Özsoy et al., 2022; Mangal and Tiwari, 2020; De Paolis and De Luca, 2020; Zulkarnain et al., 2017; Kuster et al., 2016) did not describe the patient conditions and time intervals.

**TABLE 5:** Reliability assessments of the reviewed studies using the consensus-based standards for the selection of health measurement instruments (COSMIN) checklist.

Items	First author (publish year)								
	Kuster et al. (2016)	Zulkarnian et al. (2017)	Cai et al. (2019)	Çubukçu et al. (2020)	De Paolis and De Luca (2020)	Foreman and Engsberg (2020)	Mangal and Tiwari (2020)	Koontz et al. (2022)	Özsoy et al. (2022)
1. Stable in time between repeat	D	D	A	A	D	VG	D	A	D
2 appropriate time interval	D	D	VG	A	D	VG	D	VG	D
3 measurement condition similarity	D	A	VG	VG	A	A	D	A	A
4 measure without knowledge of score	A	A	A	A	A	A	A	A	A
5 score without knowledge of score	A	A	A	A	A	A	A	A	A
6 important flaw	D	D	VG	VG	VG	VG	VG	VG	VG
7 For continuous score: ICC	IA	IA	VG	VG	IA	A	VG	VG	A
8 For ordinal score: (weighted) kappa	NA	NA	NA	NA	NA	NA	NA	NA	NA
9 For dichotomous/nominal score: Kappa	NA	NA	NA	NA	NA	NA	NA	NA	NA
**Overall Score**	**IA**	**IA**	**A**	**A**	**IA**	**A**	**D**	**A**	**D**

Abbreviations: VG, very good; A, adequate; D, doubtful; IA, inadequate; NA, not applicable.

Of the nine studies assessed for measurement errors (Table 6), four (Koontz et al., 2022; Foreman and Engsberg, 2020; Çubukçu et al., 2020; Cai et al., 2019) were rated very good or adequate, four (Özsoy et al., 2022; De Paolis and De Luca, 2020; Zulkarnain et al., 2017; Kuster et al., 2016) were rated doubtful, and one (Mangal and Tiwari, 2020) was rated inadequate. Additionally, one study (Mangal and Tiwari, 2020) did not elucidate the standard error of measurement (SEM), smallest detectable change (SDC), LOA, or coefficient of variation (COV), and five studies (Özsoy et al., 2022; Mangal and Tiwari, 2020; De Paolis and De Luca, 2020; Zulkarnain et al., 2017; Kuster et al., 2016) did not describe the patient conditions and time intervals.

**TABLE 6:** Measurement error assessments of the reviewed studies using the consensus-based standards for the selection of health measurement instruments (COSMIN) checklist.

Items	First author (publish year)									
	Kuster et al. (2016)	Zulkarnian et al. (2017)	Cai et al. (2019)	Çubukçu et al. (2020)	De Paolis and De Luca, 2020	Foreman and Engsberg (2020)	Mangal and Tiwari (2020)	Koontz et al. (2022)	Özsoy et al. (2022)	
1 stable in time between the repeat	D	D	A	A	D	VG	D	A	D	
2 appropriate time interval	D	D	VG	A	D	VG	D	VG	D	
3 similar measurement conditions	D	A	VG	VG	A	A	D	A	A	
4 measure without knowledge of score	A	A	A	A	A	A	A	A	A	
5 assign scores without knowledge of score	A	A	A	A	A	A	A	A	A	
6 important flaw	D	D	VG	VG	VG	VG	VG	VG	VG	
7 For continuous score: SEM, SDC, LOA, CV	VG	IA	VG	VG	VG	VG	IA	VG	VG	
8 For dichotomous/nominal: percentage	NA	NA	NA	NA	NA	NA	NA	NA	NA	
**Overall score**	**D**	**IA**	**A**	**A**	**D**	**A**	**IA**	**A**	**D**	

Abbreviations: VG: very good; A: adequate; D: doubtful; IA: inadequate; NA: not applicable.

Of the 12 studies assessed using the criterion validity checklist (Table 7), ten studies (Gauci et al., 2023; Jo et al., 2022; Özsoy et al., 2022; Faity et al., 2022; Mangal and Tiwari, 2020; Foreman and Engsberg, 2020; Cai et al., 2019; Xu et al., 2017; Zulkarnain et al., 2017; Kuster et al., 2016) were rated very good or adequate and two (Çubukçu et al., 2020; Muaremi et al., 2019) were rated doubtful. Additionally, one study (Çubukçu et al., 2020) did not assess the validity using correlation or area under the curve (AUC), and two studies (Çubukçu et al., 2020; Muaremi et al., 2019) did not specify the measurement details.

**TABLE 7:** Criterion validity assessments of the reviewed studies using the consensus-based standards for the selection of health measurement instruments (COSMIN) checklist.

Items	First author (publish year)											
	Kuster et al. (2016)	Xu et al. (2017)	Zulkarnian et al. (2017)	Cai et al. (2019)	Muaremi et al. (2019)	Çubukçu et al. (2020)	Foreman and Engsberg (2020)	Mangal and Tiwari (2020)	Faity et al. (2022)	Jo et al. (2022)	Özsoy et al. (2022)	Gauci et al. (2023)
1. For continuous scores: correlation or area under curve	VG	VG	VG	VG	VG	D	VG	VG	VG	VG	VG	VG
2. For dichotomous scores: sensitivity and specificity	NA	NA	NA	NA	NA	NA	NA	NA	NA	NA	NA	NA
3. Important flaw	VG	VG	VG	VG	D	D	VG	VG	VG	VG	VG	VG
**Overall score**	**VG**	**VG**	**VG**	**VG**	**D**	**D**	**VG**	**VG**	**VG**	**VG**	**VG**	**VG**

Abbreviations: VG, very good; A, adequate; D, doubtful; IA, inadequate; NA, not applicable.

## 4 Discussion

The advancement of RGB-D camera technology has enhanced single-camera markerless motion-capture systems, leading to their application in various fields such as fitness (Rica et al., 2020), sports (Tsukamoto and Sumi, 2017), and digital therapeutics (Choi et al., 2019). However, in medical contexts, these systems must demonstrate high reliability and validity, as repeated assessments are crucial for evaluating joint functions throughout treatment (Sabari et al., 1998). The shoulder joint’s complexity and extensive range of motion (ROM) (Hayes et al., 2001) present challenges in obtaining reliable and valid measurements. This study systematically reviewed studies that have measured the reliability and validity of single-camera motion-capture systems in measuring shoulder ROM.

Intra-rater reliabilities findings across six studies were inconsistent. While some studies reported excellent reliability, many indicated poor to moderate results, suggesting that fully trusting these systems in clinical practice is currently challenging. Notably, simple movements yielded relatively better reliability and less heterogeneity. Studies utilizing the Azure Kinect device reported comparatively favorable outcomes, indicating a need for further research with the lasted devices.

Validity assessments across twelve studies also showed inconsistency. Measurements of abduction and flexion demonstrated better validity compared to rotational movements. Studies focusing on simple movements reported good to excellent validity, particularly for abduction and flexion, with most exhibiting excellent validity. Extension and adduction, sharing the same anatomical plane as flexion and abduction, were often measured together in some studies (Cai et al., 2019; Xu et al., 2017); thus, they likely yield similar results. However, as no study has independently measured extension and adduction movements, further research is required on these movements.

Studies measuring simple movements exhibited significantly lower heterogeneity in both reliability and validity. Those employing correlation analyses reported no poor reliability results and only one poor validity result (Özsoy et al., 2022). In contrast, studies assessing complex movements consistently yielded poor results. This aligns with previous findings that unregulated movements increase result variability when using Kinect systems (Lee et al., 2015).

Therefore, while current single-camera motion-capture systems with RGB-D sensors may not be suitable for complex motion measurements in clinical settings, they could potentially replace traditional goniometers and motion analysis systems for assessing simple movements like flexion and abduction. However, due to relatively poor reliability results even for simple movements, these measurements cannot be considered completely reliable. Advancements in device accuracy, both in software and hardware, are necessary to improve reliability and facilitate clinical application.

Recent devices, such as Azure Kinect and RealSense, have demonstrated relatively stable results compared to earlier models like Kinect V2. Given the limited number of studies involving these newer devices and the lack of evaluations for complex movements, generalizing these findings is difficult. However, considering that Azure Kinect performed better than Kinect V2 in rotation measurements under similar conditions (Özsoy et al., 2022), further research on these latest devices is warranted.

Some studies adjusted factors such as camera distance (Cai et al., 2021), orientation (Cai et al., 2021; Xu et al., 2017) and participant posture (Kuster et al., 2016) during measurements. According to Cai et al. (2021), changing the camera distance from 1.5 to 3 m under the same conditions resulted in negligible differences in measurements. However, changing the orientation improved the measurement results from the opposite side of the object. Nevertheless, Xu et al. (2017) reported better measurement results from the center than from the left when evaluating the right hand. Cai et al. (2021) attributed this difference to body occlusion during functional tasks, which reduces accuracy. These findings suggest that optimal sensor positioning depends on the type of movement rather than distance, and that sensors should be placed to minimize body occlusion. Kuster et al. (2016) evaluated both seated and standing measurement validities, and although they recommended seated measurements, this recommendation was based on a low trunk motion bias; hence, it cannot be applied to the shoulder. Therefore, further research is required to determine the most suitable postures for shoulder ROM measurement.

Quality assessments using the COSMIN checklist revealed that the methodological quality of reliability and criterion validity ranged from inadequate to very good, while measurement error ranged from adequate to doubtful. Five studies were rated doubtful for reliability due to insufficient consideration of participants’ condition (item 1) and time interval (item 2), essential to prevent fatigue and recall bias. Although some studies used 7–21 days intervals, only a few addressed fatigue management with breaks, while others omitted participant condition details. Three studies (De Paolis and De Luca, 2020; Zulkarnain et al., 2017; Kuster et al., 2016) were rated inadequate due to the absence of ICC in their results (Item 8). Since Pearson or Spearman correlations do not reflect systematic differences between repeated measurements, ICC is preferred for continuous scores (Mokkink et al., 2021). The COSMIN checklist results for studies on measurement errors were similar to those on reliability because measurement errors and reliability are closely related. Three studies (Mangal and Tiwari, 2020; Zulkarnain et al., 2017; Kuster et al., 2016) were rated inadequate or doubtful due to the lack of SEM, SDC, LOA, and CV results (Item 7).

For criterion validity, one study (Çubukçu et al., 2020) was rated doubtful because they did not report a correlation or AUC (Item 1). Correlation is preferred when examining criterion validity because it provides information about the strength and direction of the relationship between the metric and the criterion, whereas mean bias only provides the average difference. The Bland–Altman plot can visually represent the differences between variables, but it is disadvantageous for comparing several studies. Two studies (Çubukçu et al., 2020; Muaremi et al., 2019) were rated doubtful due to the lack of measurement details (Item 3). Because ROM measurements vary among studies, and these differences can lead to different results, it is important to clearly display details such as the posture and movement used for measurements. Therefore, future studies must consider the participants’ state and break intervals to provide details on the measurement methods, and employ desirable values.

A previous systematic review on shoulder ROM measurements with Kinect (Beshara et al., 2021) evaluated only reliability and emphasized that Kinect exhibited higher reliability than inertial sensors, smartphones, and digital inclinometers. However, the results were inconsistent, with some studies indicating good intra-rater reliability and others reporting poor reliability, similar to our findings. Our study also revealed inconsistencies in validity scores among current studies and identified factors contributing to these inconsistencies, such as the complexity and direction of movements.

This study has several limitations. First, the data synthesis was limited due to the heterogeneity of the studies. While ICC is preferred for reliability and correlation, and the AUC is preferred for validity, the reviewed studies employed various metrics, making it impossible to synthesize all results. Our data synthesis only included studies that correlation analyses; therefore, the results cannot be generalized. Second, the single-camera markerless motion-capture system using an RGB-D sensor comprises both camera sensor and software. However, the effect of the software was not assessed due to its complexity and the lack of corresponding information in the studies. Future research should analyze the software used, as changes in its settings can significantly alter measurement reliability and validity. Finally, different studies used various reference standards for measuring validity, making direct comparisons difficult. Some studies employed motion analysis systems, while others used different types of goniometers. These differences may have affected the results due to variations in test methods. Therefore, further well-designed studies are required to address these limitations.

In addition to the current limitations, future research should investigate broader applications of markerless motion-capture systems. Recent technological advances now allow for the capture of complex kinematic parameters such as three-dimensional joint trajectories, angular velocity, and inter-joint coordination (e.g., scapulothoracic rhythm, compensatory trunk movement), which may improve diagnostic precision and enable more comprehensive functional assessments. For example, one study (Lee et al., 2021) has shown that shoulder disorders such as adhesive capsulitis are associated with altered angular velocities and delayed time-to-motion onset during abduction and adduction. These emerging metrics could supplement traditional ROM measures and lead to more individualized evaluations. Additionally, recent developments in vision-based mobile applications (Leung et al., 2024) suggest the potential for scalable, accessible tools for remote musculoskeletal monitoring and early disease detection. While single-camera markerless motion-capture systems have shown acceptable reliability for assessing simple, planar shoulder movements, their inherent limitations in capturing complex three-dimensional kinematics should be acknowledged. Overcoming these constraints may require integration of multi-sensor arrays or the development of more advanced computer vision algorithms.

To our knowledge, this is the first systematic review to concurrently evaluate the reliability and validity of single-camera markerless motion-capture system for shoulder ROM measurement. This review provides a comprehensive synthesis of heterogeneous evidence, stratifying findings based on movement complexity, and reinforces methodological rigor through the application of the COSMIN tool, thereby enhancing the clinical utility of its conclusions.

In conclusion, this systematic review indicates that single-camera markerless motion-capture systems utilizing RGB-D sensors hold promise for measuring shoulder joint range of motion (ROM). However, the current body of research reveals inconsistencies in reliability and validity, particularly concerning complex movements, which raises concerns about their immediate clinical applicability. Notably, measurements of simple movements such as flexion and abduction have demonstrated sufficient validation for potential clinical use. We anticipate that future high-quality studies employing more advanced devices will address these limitations, thereby enabling accurate and reliable assessments across all types of shoulder movements.

## Data Availability

The original contributions presented in the study are included in the article/Supplementary Material, further inquiries can be directed to the corresponding author.
